# Unveiling the landscape of cytokine research in glioma immunotherapy: a scientometrics analysis

**DOI:** 10.3389/fphar.2023.1333124

**Published:** 2024-01-08

**Authors:** Hongyu Zhang, Ying Chen, Xinzhan Jiang, Qiang Gu, Jiahao Yao, Xuefeng Wang, Jianghua Wu

**Affiliations:** ^1^ Department of Neurosurgery, The Fourth Affiliated Hospital of Harbin Medical University, Harbin, China; ^2^ Gamma Knife Center, Department of Oncology, Department of Neurological Surgery, Tianjin Huanhu Hospital, Tianjin Medical University, Tianjin, China; ^3^ Department of Neurobiology, Harbin Medical University, Harbin, China; ^4^ School of Nursing, Shandong First Medical University and Shandong Academy of Medical Sciences, Taian, Shandong, China

**Keywords:** cytokine, glioma, immunotherapy, scientometrics, immune microenvironment, citespace, VOSviewer

## Abstract

**Background:** Cytokines modulate the glioma tumor microenvironment, influencing occurrence, progression, and treatment response. Strategic cytokine application may improve glioma immunotherapy outcomes. Gliomas remain refractory to standard therapeutic modalities, but immunotherapy shows promise given the integral immunomodulatory roles of cytokines. However, systematic evaluation of cytokine glioma immunotherapy research is absent. Bibliometric mapping of the research landscape, recognition of impactful contributions, and elucidation of evolutive trajectories and hot topics has yet to occur, potentially guiding future efforts. Here, we analyzed the structure, evolution, trends, and hotspots of the cytokine glioma immunotherapy research field, subsequently focusing on avenues for future investigation.

**Methods:** This investigation conducted comprehensive bibliometric analyses on a corpus of 1529 English-language publications, from 1 January 2000, to 4 October 2023, extracted from the Web of Science database. The study employed tools including Microsoft Excel, Origin, VOSviewer, CiteSpace, and the Bibliometrix R package, to systematically assess trends in publication, contributions from various countries, institutions, authors, and journals, as well as to examine literature co-citation and keyword distributions within the domain of cytokines for glioma immunotherapy. The application of these methodologies facilitated a detailed exploration of the hotspots, the underlying knowledge structure, and the developments in the field of cytokines for glioma immunotherapy.

**Results:** This bibliometric analysis revealed an exponential growth in annual publications, with the United States, China, and Germany as top contributors. Reviews constituted 17% and research articles 83% of total publications. Analysis of keywords like “interleukin-13,” “TGF-beta,” and “dendritic cells” indicated progression from foundational cytokine therapies to sophisticated understanding of the tumor microenvironment and immune dynamics. Key research avenues encompassed the tumor microenvironment, epidermal growth factor receptor, clinical trials, and interleukin pathways. This comprehensive quantitative mapping of the glioma immunotherapy cytokine literature provides valuable insights to advance future research and therapeutic development.

**Conclusion:** This study has identified remaining knowledge gaps regarding the role of cytokines in glioma immunotherapy. Future research will likely focus on the tumor microenvironment, cancer vaccines, epidermal growth factor receptor, and interleukin-13 receptor alpha 2. Glioma immunotherapy development will continue through investigations into resistance mechanisms, microglia and macrophage biology, and interactions within the complex tumor microenvironment.

## 1 Introduction

Glioma, categorized as one of the most common and aggressive brain tumors, persistently confronts current medical frameworks in diagnosis, treatment, and prognostication (Liu et al., 2018). Despite substantial advancements in surgical techniques, radiation therapy, and chemotherapy, the median survival duration for patients with high-grade glioma remains limited to approximately 15 months (Hover et al., 2016). This urgent context underscores the imperative to explore innovative therapeutic modalities. Immunotherapy, leveraging the body’s immune system to detect and eradicate tumor cells, has shown promising results in a range of cancers and is increasingly being recognized as a viable therapeutic strategy in the management of glioma (Deng et al., 2014; van der Zanden et al., 2020).

Cytokines, a group of small protein molecules synthesized by immune cells, are critical in maintaining immune equilibrium and modulating pathophysiological processes in diseases such as cancer and autoimmune disorders. These molecules are categorized into various subtypes, including chemokines, interferons (IFNs), interleukins (ILs), and tumor necrosis factors (TNFs), distinguished by their receptor structures. Given their profound impact on the immune response, numerous cytokines have been identified as promising therapeutic agents in oncology. In this context, several cytokines, such as recombinant IL-2 (Sahin et al., 2020), IFNs (Tran et al., 2023), and TNFs (Gong et al., 2018; Zhu et al., 2018), have garnered clinical approval for use in immunotherapy. Their pivotal role in facilitating intercellular communication within the immune system underscores their potential in developing treatments for glioma. As research in this field expands, a thorough assessment of the academic landscape becomes imperative to identify prevailing trends, acknowledge key contributors, and unveil existing research gaps.

Bibliometrics, a methodological cornerstone for the quantitative evaluation of scientific literature (Aria and Cuccurullo, 2017), provides invaluable perspectives on the progression and dominant tendencies within specific research fields (Durieux and Gevenois, 2010; Sugimoto et al., 2019). The integration of visualization tools significantly enhances the ability of researchers to unravel complex relationships and trends within extensive data sets. Recently, there has been a marked increase in the application of bibliometric methods across diverse medical research disciplines. In oncology, for example, a multitude of studies have employed bibliometric analysis to delineate research emphases in various tumor types (Shi et al., 2021; Liu et al., 2022; He et al., 2023). In the realm of immunotherapy, these techniques have been extensively utilized to chart the development of immunotherapeutic strategies for a range of diseases (Shen et al., 2022; Zhong et al., 2022; Chen et al., 2023; Zhang et al., 2023). Although there are focused bibliometric studies on individual cytokines (Cai et al., 2019; Qin et al., 2022; Jin et al., 2023), a notable gap exists in comprehensive investigations examining the collective evolution of inflammatory mediators in glioma immunotherapy. To bridge this gap, our study leverages the combined power of bibliometric and visualization analyses to map out the research landscape surrounding cytokines in glioma immunotherapy (CGI), identifying key contributors, seminal works, and emerging trends. This initiative is of paramount importance for researchers and clinicians seeking to comprehend the current research environment and pinpoint prospective areas of investigation. Our goal is to stimulate further research, foster interdisciplinary collaboration, and accelerate therapeutic advancements in glioma treatment by offering a thorough understanding of this evolving sector.

## 2 Materials and methods

### 2.1 Search strategies and dataset establishment

**FIGURE 1:**
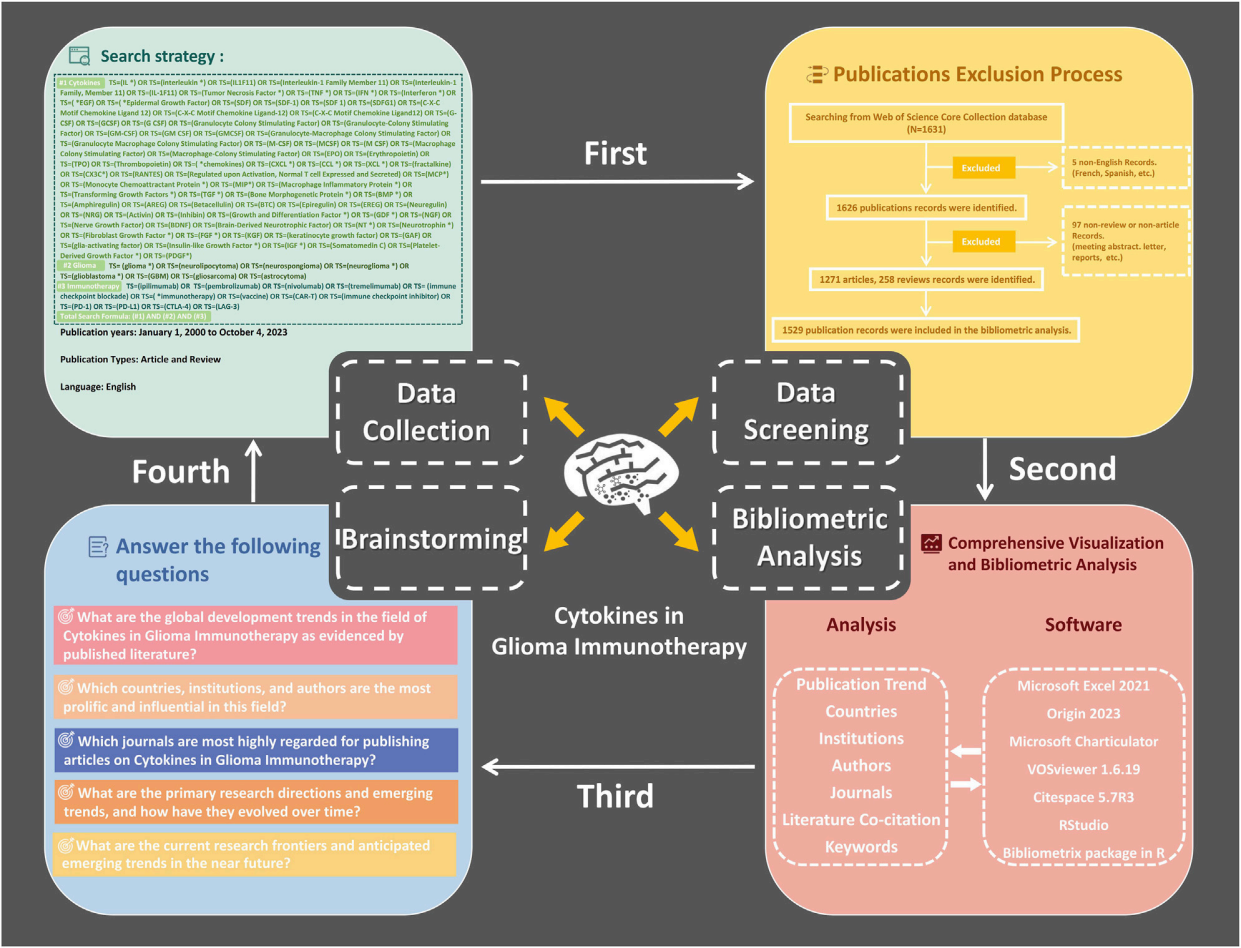
Flow diagram of this study.

The Web of Science (WoS), a product of Clarivate Analytics, stands as a preeminent academic database, hosting in excess of 12,000 high-impact journals (Birkle et al., 2020). Recognized as the gold standard for bibliometric research, the WoS core collection facilitates extensive scholarly analyses. In our investigation of CGI, a methodical and exhaustive search strategy was implemented, encapsulated in the formula: total search equation = (#1 Cytokines) AND (#2 Glioma) AND (#3 Immunotherapy). The intricacies of this search methodology are delineated in Figure 1 and Table 1.

**TABLE 1:** Retrieval formula of this bibliometric analysis.

	Sections	Search formulas
#1	Cytokines	TS=(IL *) OR TS=(interleukin *) OR TS=(IL1F11) OR TS=(Interleukin-1 Family Member 11) OR TS=(Interleukin-1 Family, Member 11) OR TS=(IL-1F11) OR TS=(Tumor Necrosis Factor *) OR TS=(TNF *) OR TS=(IFN *) OR TS=(Interferon *) OR TS=(*EGF) OR TS=(*Epidermal Growth Factor) OR TS=(SDF) OR TS=(SDF-1) OR TS=(SDF 1) OR TS=(SDFG1) OR TS=(C-X-C Motif Chemokine Ligand 12) OR TS=(C-X-C Motif Chemokine Ligand-12) OR TS=(C-X-C Motif Chemokine Ligand12) OR TS=(G-CSF) OR TS=(GCSF) OR TS=(G CSF) OR TS=(Granulocyte Colony Stimulating Factor) OR TS=(Granulocyte-Colony Stimulating Factor) OR TS=(GM-CSF) OR TS=(GM CSF) OR TS=(GMCSF) OR TS=(Granulocyte-Macrophage Colony Stimulating Factor) OR TS=(Granulocyte Macrophage Colony Stimulating Factor) OR TS=(M-CSF) OR TS=(MCSF) OR TS=(M CSF) OR TS=(Macrophage Colony Stimulating Factor) OR TS=(Macrophage-Colony Stimulating Factor) OR TS=(EPO) OR TS=(Erythropoietin) OR TS=(TPO) OR TS=(Thrombopoietin) OR TS=(*chemokines) OR TS=(CXCL *) OR TS=(CCL *) OR TS=(XCL *) OR TS=(fractalkine) OR TS=(CX3C*) OR TS=(RANTES) OR TS=(Regulated upon Activation, Normal T cell Expressed and Secreted) OR TS=(MCP*) OR TS=(Monocyte Chemoattractant Protein *) OR TS=(MIP*) OR TS=(Macrophage Inflammatory Protein *) OR TS=(Transforming Growth Factors *) OR TS=(TGF *) OR TS=(Bone Morphogenetic Protein *) OR TS=(BMP *) OR TS=(Amphiregulin) OR TS=(AREG) OR TS=(Betacellulin) OR TS=(BTC) OR TS=(Epiregulin) OR TS=(EREG) OR TS=(Neuregulin) OR TS=(NRG) OR TS=(Activin) OR TS=(Inhibin) OR TS=(Growth and Differentiation Factor *) OR TS=(GDF *) OR TS=(NGF) OR TS=(Nerve Growth Factor) OR TS=(BDNF) OR TS=(Brain-Derived Neurotrophic Factor) OR TS=(NT *) OR TS=(Neurotrophin *) OR TS=(Fibroblast Growth Factor *) OR TS=(FGF *) OR TS=(KGF) OR TS=(keratinocyte growth factor) OR TS=(GAF) OR TS=(glia-activating factor) OR TS=(Insulin-like Growth Factor *) OR TS=(IGF *) OR TS=(Somatomedin C) OR TS=(Platelet-Derived Growth Factor *) OR TS=(PDGF*)
#2	Glioma	TS= (glioma *) OR TS=(neurolipocytoma) OR TS=(neurospongioma) OR TS=(neuroglioma *) OR TS=(glioblastoma *) OR TS=(GBM) OR TS=(gliosarcoma) OR TS=(astrocytoma)
#3	Immunotherapy	TS=(ipilimumab) OR TS=(pembrolizumab) OR TS=(nivolumab) OR TS=(tremelimumab) OR TS= (immune checkpoint blockade) OR TS=(*immunotherapy) OR TS=(vaccine) OR TS=(CAR-T) OR TS=(immune checkpoint inhibitor) OR TS=(PD-1) OR TS=(PD-L1) OR TS=(CTLA-4) OR TS=(LAG-3)
Total Search Formula		(#1) AND (#2) AND (#3)

The inclusion criteria for source selection in this research were defined as follows: 1) Manuscripts must explicitly address the topic of CGI and be fully accessible; 2) Literature must be published within the timeframe of 1 January 2000, to 4 October 2023; 3) Only documents categorized as “articles” or “reviews” were considered; and 4) All publications had to be in English. The exclusion criteria were: 1) Articles not directly related to CGI; 2) Other document formats such as letters, reports, short communications, abstracts, et al.; 3) Duplicate studies. Two investigators, HZ and XJ, independently evaluated each publication for compliance with these criteria. Any disagreements were reconciled through consultative discussions with co-authors, ensuring the precision of data curation. The comprehensive screening methodology is illustrated in Figure 1. Employing this systematic approach, a total of 1,529 publications were extracted from the Web of Science, as detailed in Supplementary Table S9.

### 2.2 Bibliometric analysis and visualization

This study entailed a bibliometric analysis to scrutinize the corpus of literature pertaining to CGI across multiple facets: temporal publication trends, contributions by countries, institutions, authors, journals, literature co-citation, and keyword prevalence. A suite of software tools was employed for this comprehensive analysis: Microsoft Excel 2021, Origin 2023, Microsoft Charticulator, VOSviewer 1.6.19, Citespace 5.7R3, and the Bibliometrix package within RStudio. Specific aspects of the analysis included: examining annual publication trends using Excel and Origin; assessing country-level research contributions with Excel, Charticulator, VOSviewer, and Bibliometrix; institutional activities were analyzed via Excel and Citespace; author contributions were evaluated using VOSviewer, Bibliometrix, and Excel; journal publication metrics were derived using Origin, VOSviewer, Citespace, and Bibliometrix; co-citation networks were mapped using Citespace; and keyword trends were analyzed through VOSviewer and Citespace.

Within the visual network diagrams, nodes symbolize various parameters, including countries, institutions, or keywords. The dimension of each node is indicative of its relative importance, with larger nodes signifying greater prominence. Both nodes and their interconnecting lines are distinguished by distinct color schemes, corresponding to specific clusters. The spatial proximity between nodes is representative of the intensity of co-authorship or co-citation links, wherein thicker lines signify more robust connections.

## 3 Results

### 3.1 Analysis of publication trend

**FIGURE 2:**
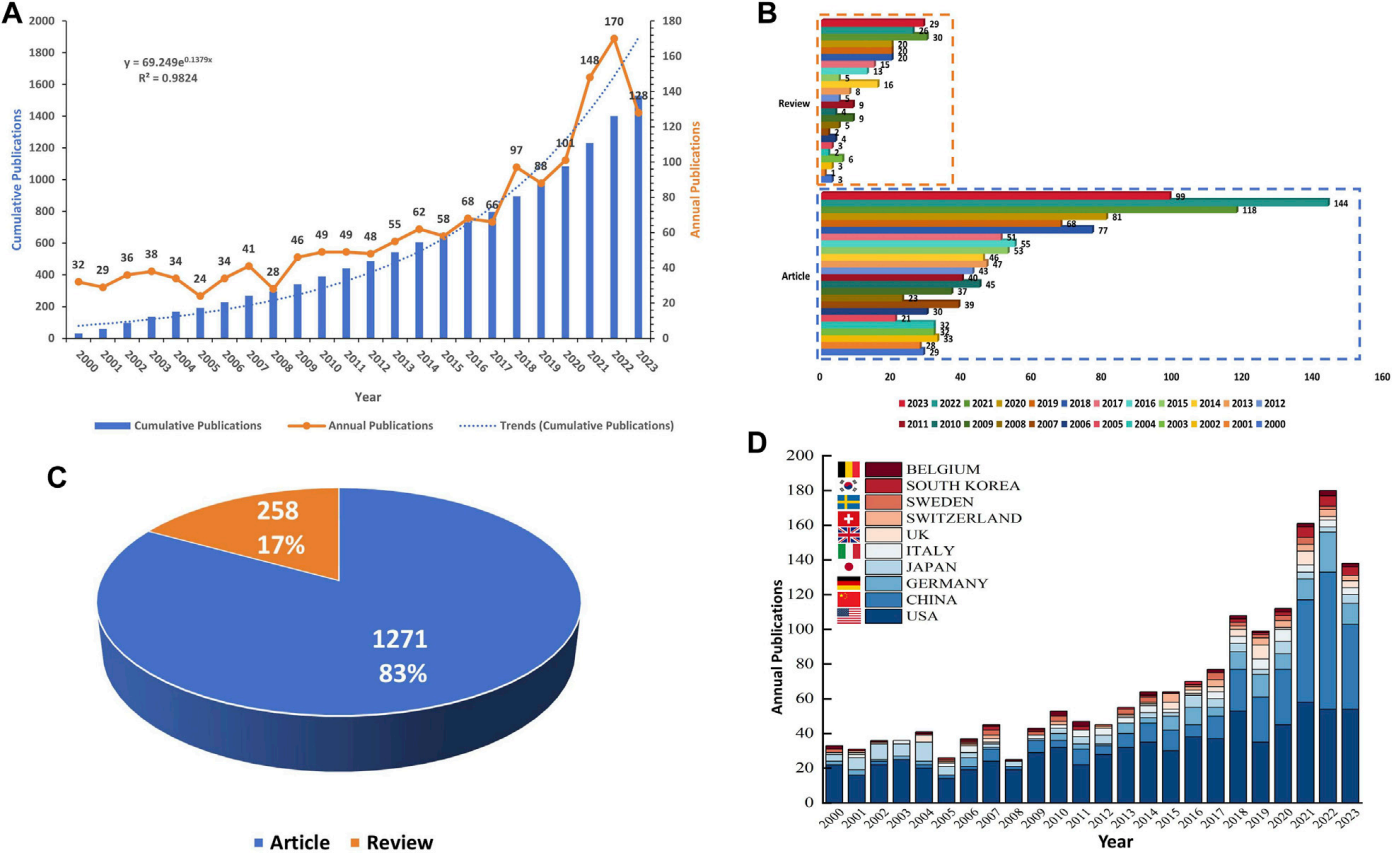
**(A)** Global publication trends from 1 January 2000, to 4 October 2023 on CGI; **(B)** Bar graph of the annual volume of reviews and articles published during the aforementioned period; **(C)** Pie chart of the proportion of reviews and articles in this field; **(D)** Bar graph of annual publications from the top ten countries in the field. Abbreviation: CGI, cytokines in glioma immunotherapy.

This bibliometric investigation encompassed an analysis of 1,529 papers on CGI, published across 433 journals, originating from 1,778 institutions in 49 countries. The temporal distribution of these publications, as depicted in Figure 2A, illustrates a fluctuating pattern from 2000 to 2019, with the annual publication count initially falling below 25 in 2005. Although there was a steady increase in yearly publications throughout this period, the count did not exceed 100. This trend indicates a relatively limited focus on this area by researchers during these years. However, a pronounced surge in the annual number of publications was observed from 2019 to 2023. In 2020, the count exceeded 100 for the first time, reaching 101, and further escalated to 170 by 2022. The dotted line in the figure represents the total number of publications over this period. An exponential time prediction curve model was applied to this data, yielding the formula y = 69.249e^0.1379x^. This model demonstrated a statistically significant correlation (*R* ^2^ = 0.9824) between the year and the cumulative number of publications, suggesting a strong fit. The cumulative growth rate of publications was modest from 2000 to 2016 but became more pronounced from 2016 to 2023. By 2022, the total number of publications had reached approximately 1,400. It is projected that the annual publication count in this domain might reach its zenith in 2023, a prediction contingent on the fact that 2023 has not yet concluded, and the peak may still be forthcoming.

Figures 2B,C delineate the composition of the total publications in the field of CGI, revealing that reviews account for 17% and research articles for 83%. During the period from 1 January 2020, to 4 October 2023, the highest incidence of review publications occurred in 2021, reaching a total of 30, while the year 2001 registered the lowest with only one review. In parallel, the year 2022 witnessed the zenith of article publications, numbering 144, in stark contrast to 2001, which saw the minimum at 21 articles. Figure 2D exhibits an ascending trajectory in the output of the top ten contributing countries in this domain over the past 23 years, with the United States, China, and Germany being particularly prominent. This trend underscores the escalating scholarly interest in CGI, highlighting its emerging prominence in academic research.

### 3.2 Analysis of research countries

**FIGURE 3:**
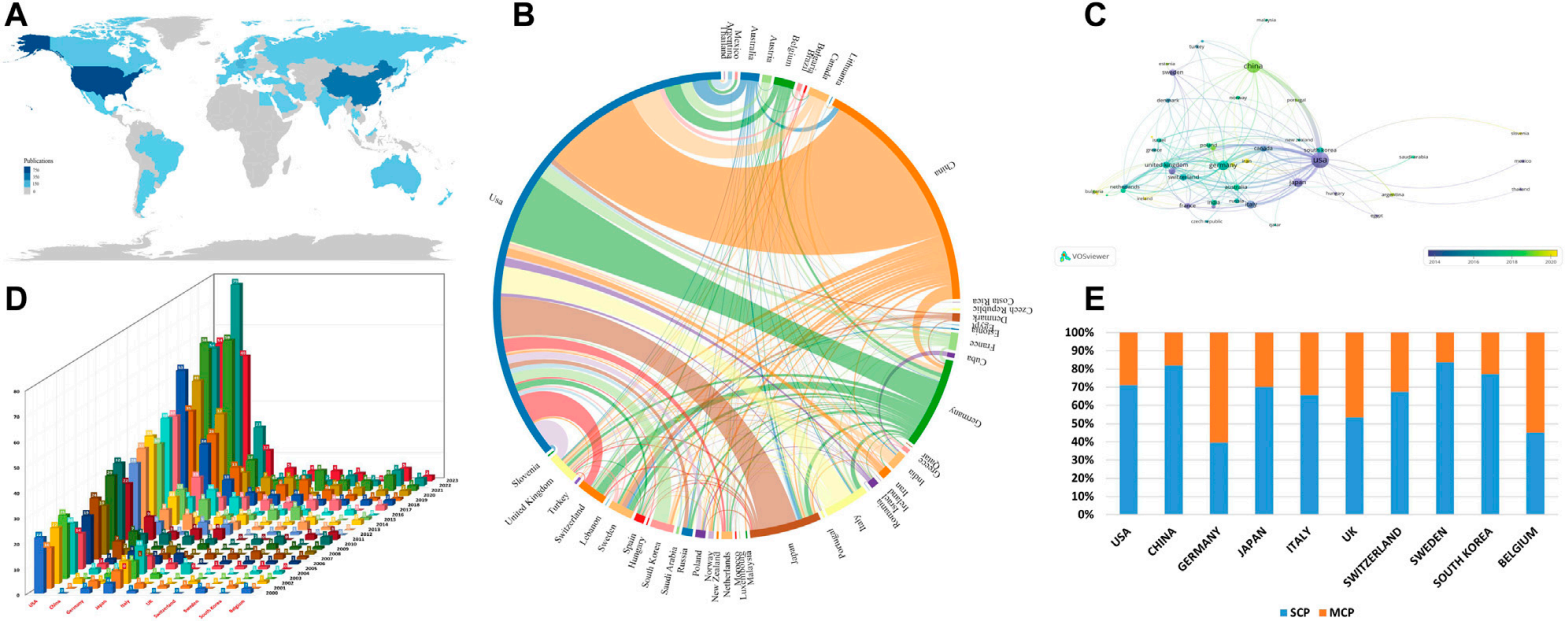
**(A)** Geographical distribution of publications worldwide; **(B)** An analysis of international collaborations among countries is provided. Connections between countries denote collaborative ties, with thicker lines signifying more robust collaborations; **(C)** A country analysis visualized using VOSviewer is displayed. Nodes represent countries, with their size correlating to the number of publications. The color gradient, from purple (earliest) to yellow (most recent), signifies the timeline of publications for each country; **(D)** Bar graph of the number of publications per year from 2000 to 2023 for the top ten countries; **(E)** The number of publications of SCP and MCP in the top 10 countries by volume. Abbreviation: SCP refers to Single Country Publications, where all authors hail from the same nation. Conversely, MCP stands for Multiple Country Publications, denoting articles with authors from different countries, signifying international collaboration.

This statistical analysis delineates the distribution and comparative evaluation of cytokine research in glioma immunotherapy across various countries, spanning from 1 January 2000, to 4 October 2023. Within this timeframe, 49 countries have engaged in this research area. The leading 10 countries in terms of publication output are predominantly developed nations, with China and South Korea as notable exceptions (Table 2). The data reveals that the United States serves as a hub of international collaboration, maintaining the most substantial cooperative ties with China. The United States and China are the foremost contributors to the literature, accounting for 49.9% and 23.7% of total publications, respectively. The upward trend in publications is especially evident in the United States, China, and Germany (Figure 3D and Supplementary Table S1). Geographically, the bulk of cytokine research in glioma immunotherapy is concentrated in North America, South America, Europe, Asia, and Oceania (Figure 3A). Figure 3B shows the United States, China, and Germany at the forefront of global collaboration, as measured by the analysis of international partnerships. Among all countries, China exhibits the most extensive collaboration with the United States, followed by Germany and Canada. Recent years have seen the emergence of new partnerships with countries like Iran, Slovenia, and Ireland, while the United States maintains longstanding collaborations (Figure 3C). Notably, only Belgium and Germany among the top ten countries have published more papers through international collaboration than domestic efforts, suggesting a need for increased international cooperation among scholars in countries with lower rates of global collaboration (Figure 3E and Supplementary Table S2). In terms of average citations per paper, Switzerland surpasses the United States, although the latter leads in total citations. China, despite being second in publication volume, has the lowest citation count among the top ten countries. However, the recent increase in international collaborations and annual publications from China since 2022 highlights its growing significance in the field, underscoring the need for Chinese researchers to focus on enhancing the quality and citation impact of their publications.

**TABLE 2:** Top 10 countries by number of publications on CGI.

Rank	Country	Number (%)	Citations	ACPP	H-index
1	United States	763 (49.9%)	44,002	57.7	105
2	China	362 (23.7%)	6,782	18.7	42
3	Germany	136 (8.9%)	6,960	51.2	40
4	Japan	107 (7.0%)	3,973	37.1	31
5	Italy	67 (4.4%)	2,654	39.6	28
6	England	45 (3.0%)	3,241	70.3	19
7	Switzerland	43 (2.8%)	3,243	75.4	26
8	Sweden	37 (2.4%)	862	23.3	16
9	South Korea	35 (2.3%)	711	20.3	14
10	Belgium	31 (2.0%)	1,565	50.5	19

Abbreviation: CGI, cytokines in glioma immunotherapy; ACPP, average citation per publication.

### 3.3 Analysis of research institutions

**FIGURE 4:**
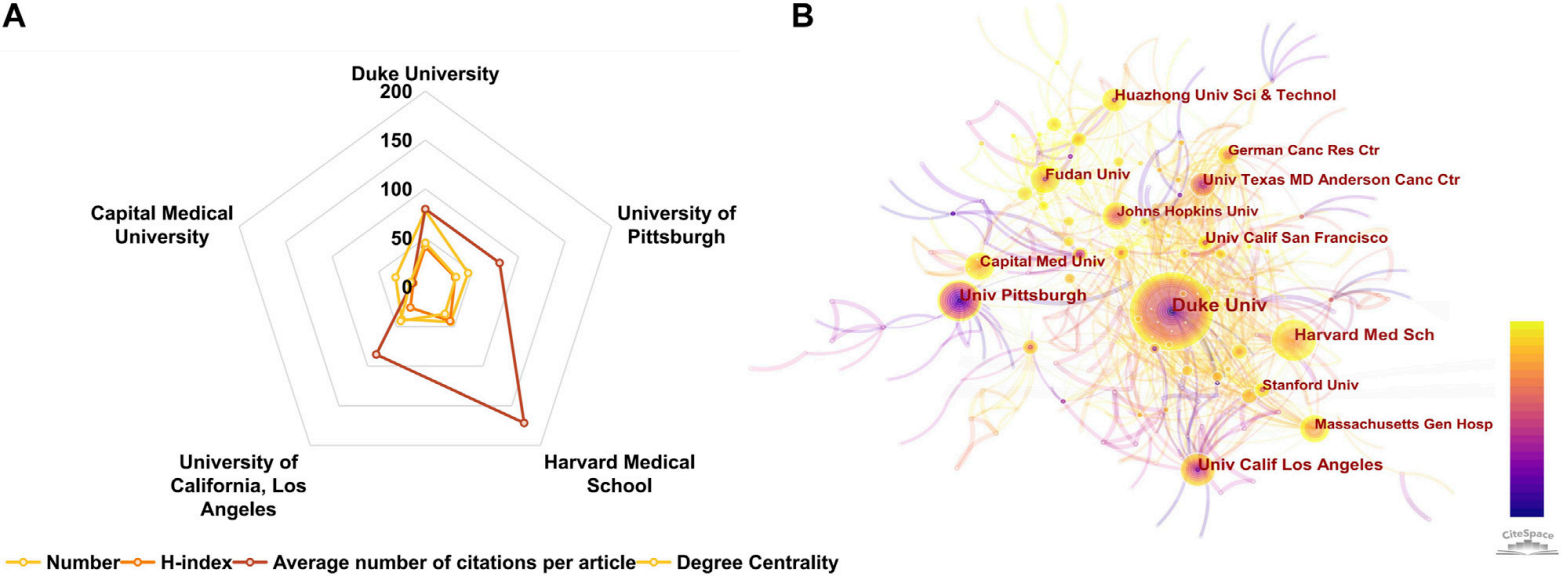
**(A)** Radar chart of the top five published institutions. **(B)** The co-occurrence map of research institutions generated by CiteSpace. Each node signifies an institution, with its size reflecting publication output. Connections between nodes indicate collaboration, with thicker lines suggesting closer partnerships. Node and line colors denote publication years, with lighter shades representing more recent years. Nodes with a centrality value greater than 0.1 are highlighted by purple rings.

Citespace was utilized to analyze the contributions of leading institutions in the field of CGI. Among the 1,529 publications reviewed, a total of 433 distinct institutions were identified as contributors. The top ten most prolific institutions are enumerated in Table 3. Within this group, three institutions are based in China, while seven are located in the United States, reflecting the overall geographic distribution of the contributing countries. Duke University emerged as the foremost institution in terms of publication volume with 78 studies, followed by the University of Pittsburgh with 46, Harvard Medical School with 44, and the University of California, Los Angeles with 41 publications. Capital Medical University in China ranked fifth in terms of publication count. The radar plots in Figure 4A and data in Supplementary Table S3 indicate that among these top five institutions, Duke University demonstrated the highest degree centrality, while Harvard Medical School exhibited the highest h-index. Degree centrality, defined by the number of connections a node has, serves as an indicator of an institution’s significance within bibliometric networks. Institutions with high degree centrality are typically well-connected and extensively cited, both within and across various research domains, denoting their influential role. Such pivotal works often mark research hotspots and contextual developments, forming crucial nodes in scholarly networks. Therefore, degree centrality is instrumental in identifying key literature to map knowledge domains and track disciplinary evolution, serving as a vital measure of academic impact. In terms of Average Citation Per Paper (ACPP), the University of Texas MD Anderson Cancer Center ranked highest, followed by Harvard Medical School, which, despite having the highest overall citation count, ranked second. This finding is congruent with the national analysis, where the United States has made significant contributions and maintained a dominant position in the field.

**TABLE 3:** The top 10 institutions ranked by number of publications on CGI.

Rank	Institutions	Number	TC	ACPP	H-index	Degree centrality	Country
1	Duke University	78	6,213	79.7	41	45	United States
2	University of Pittsburgh	46	3,668	79.7	32	33	United States
3	Harvard Medical School	44	7,553	171.7	43	34	United States
4	University of California, Los Angeles	41	3,497	85.3	26	43	United States
5	Capital Medical University	32	453	14.2	13	16	China
6	University of Texas MD Anderson Cancer Center	29	5,407	186.5	30	31	United States
7	University of California, San Francisco	28	4,019	143.5	31	35	United States
8	Huazhong University of Science and Technology	28	269	9.6	10	23	China
9	Fudan University	28	614	21.9	13	18	China
10	Johns Hopkins University	27	1,434	53.1	14	20	United States

Abbreviation: CGI, cytokines in glioma immunotherapy; TC, total citations; ACPP, average citations per paper.

An analysis of institutional cooperation was conducted to elucidate collaborative dynamics between institutions (Figure 4B). In these visualizations, nodes represent individual institutions, with the size of each node proportional to its publication output. The connecting lines between nodes signify collaborative relationships, with line thickness indicating the strength of cooperation; denser lines denote more robust collaboration. The color gradient in the nodes, transitioning from purple to yellow from 2000 to 2023, represents the centrality of each institution. Notably, Capital Medical University and Fudan University in China, along with Johns Hopkins University in the United States, demonstrated degree centralities of 16, 18, and 20, respectively, but exhibited relatively weaker connections to other institutions. In light of these findings, it is advisable for these institutions to actively pursue enhanced international cooperation, such as by engaging in global research collaborations and participating in international academic conferences, to expand their academic networks beyond their current spheres.

### 3.4 Analysis of research authors

**FIGURE 5:**
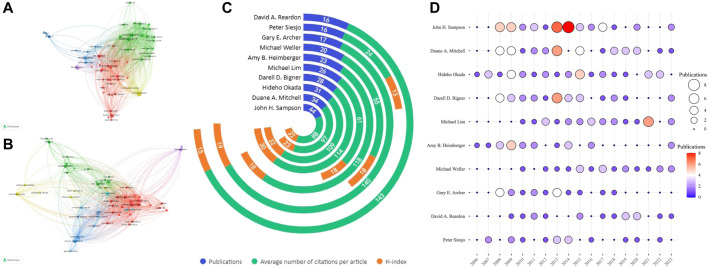
A network map depicting authors is illustrated with the following key features: **(A)** Authors with the most publications; **(B)** Most frequently cited authors. **(C)** Jade block plot of the top 10 authors with the highest number of publications in the field; **(D)** Bubble plot of the number of publications per year by the top 10 authors. The size of each circle indicates the number of publications an author has, with larger circles representing a greater number of documents. The color gradient of the circles, transitioning from purple to red, signifies an increase in the number of publications an author has in a given year.

To analyze authors in this research field, VOSviewer software, Microsoft Excel, and RStudio were used. The initial pool of 8,900 authors was narrowed to 70 by applying a threshold of at least 8 publications per author for in-depth analysis. The top 10 most prolific authors in the field were identified based on the stated criteria and are visualized in Table 4. The top 10 most-cited authors determined through co-citation analysis are presented in Table 5. Figure 5A illustrates that the majority of global partnerships involved core authors hailing from the United States, who served as the central point for multiple subgroups. Notable individuals within these subgroups include John H. Sampson, Hideho Okada, Michael Lim, and several others. Strengthening such international cooperation will likely contribute to further development of this field. Average citations per paper and h-index are key indicators for evaluating the academic influence of authors (Figure 5C). As former director of the Department of Neurosurgery at Duke University, Sampson has published 44 total articles in this field, accruing 3,861 citations to date. His H-index is 32 and total link strength 64,537, ranking first among the top ten authors by volume of publications, reflecting his outstanding contributions to the field. His primary research interests include glioblastoma treatment and immunotherapy, though he does not have the highest average citations per paper. That distinction belongs to David A. Reardon of Harvard Medical School, whose average citations per paper are 143, the highest among the top ten authors. Dr. Reardon’s research focuses on neuro-oncology, particularly treating malignant brain tumors through novel therapeutic strategies, clinical trials, immunotherapy, targeted therapies, and disease biology. Figure 5D depicts the annual publication counts of the top ten authors between 2006 and 2023. Figure 5A displays circles of varying sizes that represent the number of publications, where bigger circles indicate a higher number of publications. The color gradient, transitioning from purple to red, also represents increasing publication counts. It is evident from the data that these ten authors had a prolific output between 2013 and 2015. Specifically, in 2013, John H. Sampson, Duane A. Mitchell, and Darell D. Bigner had notably high publication output. John H. Sampson was particularly prolific in 2014, authoring 8 publications that year (Supplementary Table S4).

**TABLE 4:** Top 10 authors ranked by number of publications.

Rank	Author	Publications	ACPP	H-index	Citations	Total link strength
1	John H. Sampson	44	88	32	3,861	64,537
2	Duane A. Mitchell	34	97	23	3,295	40,881
3	Hideho Okada	31	109	32	3,373	19,470
4	Darell D. Bigner	28	114	30	3,204	32,000
5	Michael Lim	26	61	18	1,580	27,356
6	Amy B. Heimberger	23	118	18	2,713	32,131
7	Michael Weller	20	55	18	1,097	14,167
8	Gary E. Archer	17	140	19	2,380	28,958
9	Peter Siesjo	16	24	13	378	6,395
10	David A. Reardon	16	143	15	2,286	22,253

Abbreviation: ACPP, average citations per paper.

**TABLE 5:** Top 10 co-cited authors in citations.

Rank	Author	Citations	Total link strength	Country
1	John H. Sampson	3,861	1,617	United States
2	Hideho Okada	3,373	638	United States
3	Duane A. Mitchell	3,295	1,097	United States
4	Darell D. Bigner	3,204	926	United States
5	Amy B. Heimberger	2,713	941	United States
6	Gary E. Archer	2,380	865	United States
7	Behnam Badie	2,374	472	United States
8	Christine E. Brown	2,318	473	United States
9	David A. Reardon	2,286	664	United States
10	Michael C. Jensen	2,228	275	United States

Figure 5B, employing VOSviewer’s co-citation analysis, depicts the functional and thematic significance of authors cited over 100 times in the field of CGI research. This analysis formulated a network comprising 70 authors who are highly cited in this domain. In this network, each node symbolizes an author, with the node size proportional to their publication count. The thickness of the lines connecting these nodes indicates the strength of co-citation relationships between authors. Authors grouped by similar colors suggest a tendency for their works to be frequently cited together. The outcomes of this author-centric analysis align closely with the results of the co-citation analysis, showing that more prolific authors are likely to be co-cited more frequently. According to Table 5, among the top 10 cited authors, John H. Sampson emerged with the highest number of citations (3,861) and the greatest overall link strength, followed by Hideho Okada with 3,373 citations, and Duane A. Mitchell with 3,295 citations. Notably, there were active co-citation linkages observed, such as those between John H. Sampson, Amy B. Heimberger, Roger E. McLendon, and Michael Weller, as well as between Michael Lim, Jacob Ruzevick, and Anhua Wu. Analyzing these interconnected relationships provides deeper insights into the collaborative dynamics among these authors, thereby enriching the understanding of research trends and networks within this specialized field.

### 3.5 Analysis of research journals

#### 3.5.1 Research journals

**FIGURE 6:**
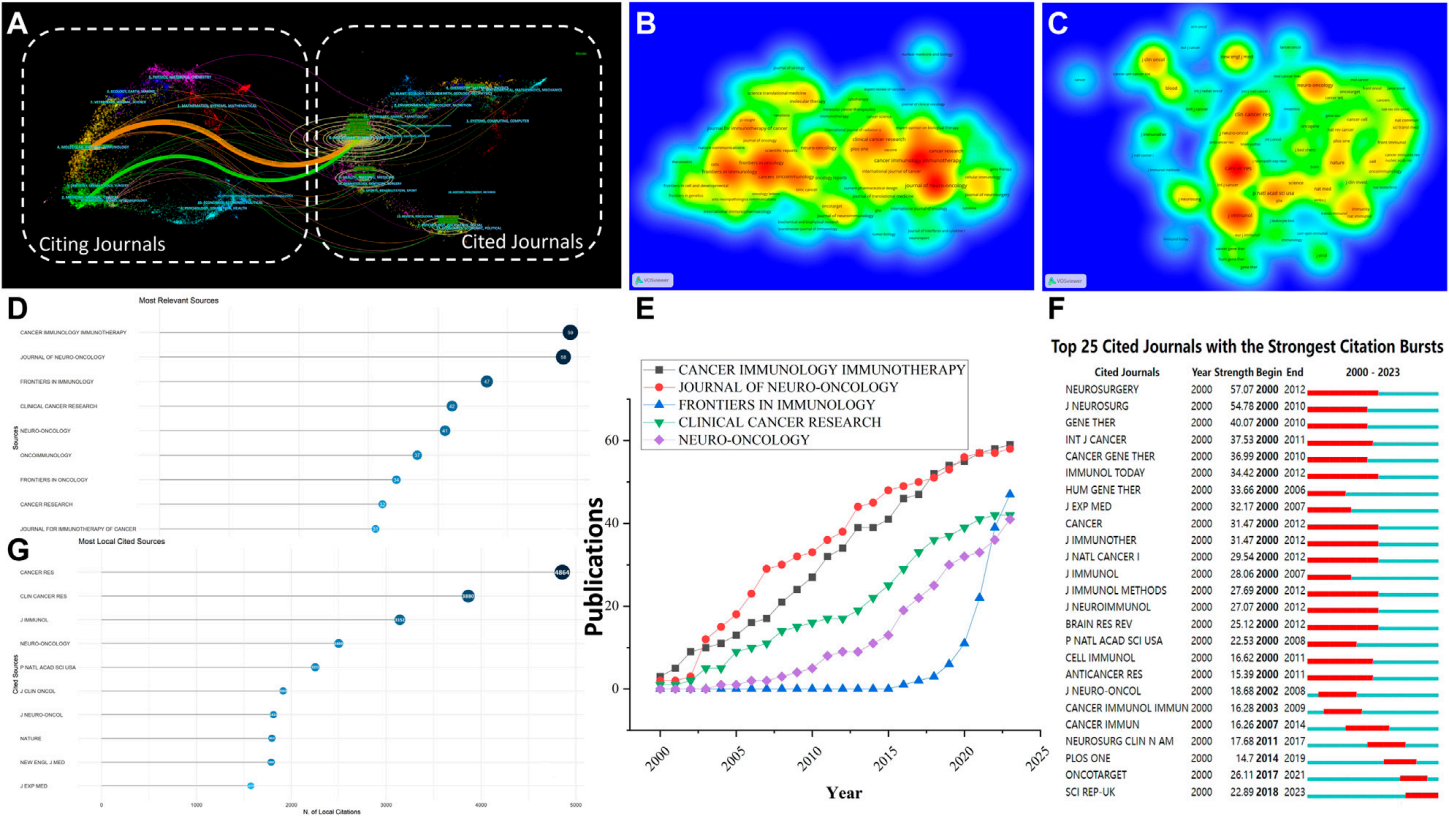
**(A)** The dual-map overlay, produced using CiteSpace, presents CGI research. On this map, the journals being cited are displayed on the right, while the citing journals are on the left. Wider lines denote predominant citing pathways; The density map of journals **(B)** and co-cited journals **(C)** about CGI research; A Cleveland dot plot presents the top 10 journals, ranked by publications **(D)** and citations **(G)**, spanning from 1 January 2000, to 4 October 2023; **(E)** Line graph of the publication trends of the top ten journals over the years; **(F)** Top 25 cited journals with the strongest citation bursts. Abbreviation: CGI, cytokines in glioma immunotherapy.

The examination of publication sources revealed the primary journals in this particular area. Table 6 and Figure 6D present the specifics of the top ten journals, which were selected according to their highest publication volume. Supplementary Table S5 presents the list of the top 10 co-cited journals obtained from citation analysis, while Figure 6G provides a visual representation of the same. Cancer Immunology Immunotherapy emerged as the journal with the highest number of publications, totaling 59, while Journal of Neuro-Oncology followed closely with 58 publications and an impact factor of 3.9. Additionally, Frontiers in Immunology contributed significantly with 47 publications and an impressive impact factor of 7.3, which corresponds to the density map depicted in Figure 6B. The Journal of Neuro-Oncology had an impact factor of 3.9, while Neuro-Oncology had an impact factor of 15.9, making them the top journals in terms of impact factors. Cancer Research ranked first among the top 10 journals with the highest number of citations (4,864), while Clinical Cancer Research (3,880) and Neuro-Oncology (2,499) followed suit, which corresponds to the density map depicted in Figure 6C. In publications on glioma, Cancer Research made significant contributions to cytokine immunotherapy, as evidenced by its highest total link strength of 419,653. Through the use of dual-map overlay analysis, distinct patterns within the worldwide scientific journal landscape were uncovered. Figure 6A generated a dual map of research on cytokine immunotherapy for glioma that was published from 2000 to 2023.The colored lines starting from the source journal collection map (on the left) and leading to the destination journal collection map (on the right) represent the trajectory of citation connections. The overlay of the biplot suggests a significant clustering of journals that focus on this subject. The majority of the source journals and references pertain to molecular biology and genetics, with the citation chain mainly confined to these fields and limited cross-disciplinary research. In the future, potential research areas in this field include physics, materials science, chemistry, veterinary science, medicine, neurology, sports science, ophthalmology, and psychology. Emerging areas of research are expected to include education and health. As can be seen from the Figure 6E and Supplementary Table S6, during the period from 2000 to 2023, *FRONTIERS IN IMMUNOLOGY* showed an obvious increase from 2018, while the other four journals showed a stable increase. For burst monitoring of journals (Figure 6F), the top three ranked journals were *NEUROSURGERY*, bursting from 2000 to 2012, followed by *JOURNAL OF NEUROSURGERY*, bursting from 2000 to 2010, and *GENE THERAPY*, bursting from 2000 to 2010.

**TABLE 6:** Top 10 Journals in terms of the number of published papers.

Rank	Journal	Number	TC	ACPP	IF	Quartile in category
1	Cancer Immunology Immunotherapy	59	2,172	36.81	5.8	Q1
2	Journal of Neuro-Oncology	58	1,965	33.88	3.9	Q2
3	Frontiers in Immunology	47	812	17.28	7.3	Q1
4	Clinical Cancer Research	42	5,145	122.50	11.5	Q1
5	Neuro-Oncology	41	2,550	62.20	15.9	Q1
6	Oncoimmunology	37	901	24.35	7.2	Q1
7	Frontiers in Oncology	34	707	20.80	4.7	Q2
8	Cancer Research	32	3,570	111.56	11.2	Q1
9	Journal for Immunotherapy of Cancer	31	917	29.58	10.9	Q1
10	Cancers	27	504	18.67	5.2	Q1

Abbreviation: TC, total citations; ACPP, average citations per paper; IF, impact factor.

#### 3.5.2 Landscape of cytokine research for glioma immunotherapy in journals with high impact factors (>20)

Several recent studies published in prestigious journals like Nature, Science Immunology, and Cancer Cell highlight promising progress in cytokine research to enhance glioma immunotherapy. Approaches include targeting inhibitory cytokines like IL-8 to potentiate immune checkpoint blockade (*Cancer Cell*), exploiting IFNγ signaling pathways to improve CAR T cell efficacy (*Nature*), using an engineered cytokine nanocarrier for deliver and tumor vessel normalization (*Nature Nanotechnology*), and modulating abnormal tryptophan metabolism via cytokines to overcome immunosuppression (*Nature Cancer*). Beyond inhibitory cytokines, other strategies aim to stimulate key cytokines to reinvigorate anti-tumor immunity, such as using CCL3 cytokine synergistically with antigens (*Nature*). Some translational insights indicate cytokines like IL-12 can enhance dendritic cell vaccine potency in glioblastoma patients (*Nature*).

While counteracting inhibitory cytokines and harnessing stimulatory cytokines show promise, an emerging frontier is targeting cytokine signaling cascades intracellularly to more potently reprogram tumor microenvironments. For example, recent work published in *Nature Cancer* revealed that inhibiting KDM6B epigenetically reprograms cytokine networks, shifting macrophages from immunosuppressive to immunostimulatory phenotypes and sensitizing gliomas to PD-1 checkpoint blockade. Similarly, directly manipulating cytokine transcription pathways, like disrupting abnormal lysine catabolism to restore histone marks, can allow cytokine effector programs to be rewritten (*Nature*). And deeper analysis of single cell cytokine networks via RNA sequencing continues to uncover opportunities for immunomodulation, with recent work delineating cytokine dynamics of tumor progression in *Nature Immunology*. Translationally, JCO (*Journal of Clinical Oncology*) papers demonstrate patient cytokine responses, as vaccines inducing CD8^+^ T cells and antigen-specific immunity in recurrent gliomas. Overall, manipulation of cytokine signaling is garnering high interest, with various innovative methodologies published in top journals demonstrating the ability of cytokines to stimulate, sustain, or restore anti-tumor immune responses. Key challenges remain in optimizing combination therapies, minimizing toxicities, and translating promising immunotherapies to clinical studies. But the field displays enthusiasm for cytokines’ potential to improve outcomes for glioma patients. Advanced intracellular targeting of cytokine signaling combined with deeper interrogation of glioma immune ecology shows immense promise to make immunotherapy more efficacious. Rational combination therapies and sequencing of cytokine modulators with existing treatments like checkpoint inhibitors could provide synergistic benefits. The breadth of innovation indicates cytokines may be key complements making immunotherapies smarter and more precise (Supplementary Table S7).

### 3.6 Analysis of research keywords

**FIGURE 7:**
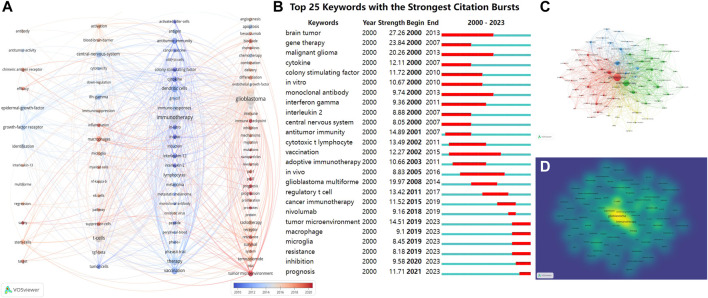
**(A)** Using VOSviewer, keywords are represented as nodes; distinct colored vertical lines indicate keyword clustering, and connections between nodes signify keyword co-occurrence. From blue to red, it represents time from the past to the present; **(B)** Top 25 cited keywords with the strongest citation bursts; **(C)** Thematic network maps depict keyword trends related to CGI research between 1 January 2000, and 4 October 2023; **(D)** The density of keyword co-occurrence is shown, with the most prevalent keywords accentuated in yellow. Abbreviation: CGI, cytokines in glioma immunotherapy.

Keywords represent the core themes of an article. Analyzing these keywords reveals the central focus of an article. To examine the co-occurrence of keywords, we employed overlay visualizations that included network and density maps. After starting with a total of 5,317 keywords, we incorporated the keyword occurrence of over 27. This criterion aimed to concentrate on the most pertinent and recurrent terms, narrowing our analysis to the 79 most frequent keywords. These were then categorized into four distinct clusters, as depicted in Figure 7A.

The network graphs primarily organized the keywords into four distinct categories. From left to right, the first column, represented research related to strategies involving the use of cytokines for targeted therapy of glioma, this group included regression, stem cells, multiforme, interleukin-13, efficacy, safety, epidermal growth factor receptor, target, antitumor activity, antibody and chimeric antigen receptor. The second column, focused on the mechanisms by which cytokines regulate the activity of immunosuppressive factors in the tumor microenvironment and tumor-infiltrating immune cells, with keywords such as Regulating immune microenvironment (inflammation, immunosuppression, macrophages, microglia, myeloid cells); Blood-brain barrier (central nervous system); Immune cell activity (activation, cytotoxicity, NK cells, T cells); Signaling pathways and transcription factors (NF-kappaB, pathway); Immunosuppressive factor (TGF-beta, suppressor cells). The third column, encompassed terms commonly associated with direct activation and enhancement of antitumor immune responses by cytokines, such as Stimulates anti-tumor immunity (antitumor immunity, immunotherapy, induction); Activating immune cells (activated killer cells, CD8^+^ T cells, dendritic cells, lymphocytes); Cytokines and cancer vaccines (cancer vaccine, cytokine, GM-CSF, interleukin-2, peptide); Clinical trial (*in vitro*, *in vivo*, phase I/II trial). The fourth column, represented research related to important players and potential targets of immunotherapy, such as angiogenesis, immune, chemokines, endothelial growth factor, proliferation, growth, differentiation, mutations, protein, combination, chemotherapy, radiotherapy and bevacizumab. The size of the circle represents how often the keyword appears, and from blue to red, the time is getting closer to the present. Upon examining the trend topics over time, it became evident that they sequentially represented the evolving understanding of CGI, mirroring the shifts in research emphasis and progression within this domain. These emergent topics indicate a shifting emphasis toward a more nuanced comprehension of the intricate interactions between tumors and the immune system. Significantly, keywords in the fourth column, including immune checkpoint, endothelial growth-factor, pd-1, pd-11, tumor microenvironment, nanoparticles, and angiogenesis, indicate a clear shift in the direction and focus of forthcoming research efforts.

Figure 7B illustrates the significant bursts of keywords identified from 2000 to 2023, portraying the evolution and trend of these keywords visually. We determined the occurrence, importance, and unexpectedness of mentioned sources (minimum duration 2). Each slice represents 1 year and references are organized based on the year the burst started. The strength-value represents the burst strength of the citation. Research hotspots that evolved over time were identified, with the earliest occurrences of “brain tumor,” “gene therapy,” “malignant glioma,” and “cytokines” indicating keyword bursts. “Prognosis,” “inhibition,” “resistance,” “microglia,” “macrophages” and “tumor microenvironment” continued into 2023.To narrow down to the most important and commonly used terms, a minimum occurrence threshold of 27 was applied from a starting set of 5,317 keywords. This refined the analysis to the 79 most common keywords, which were divided into four distinct clusters (Figure 7C). This was consistent with Figure 7A, with keyword co-occurrence density mapped based on frequency (Figure 7D). The keywords “glioblastoma” (1,213 occurrences), “immunotherapy” (803 occurrences), and “t-cells” (409 occurrences) are the most prevalent, as determined by co-occurrence density.

### 3.7 Analysis of literatures Co-citation

**FIGURE 8:**
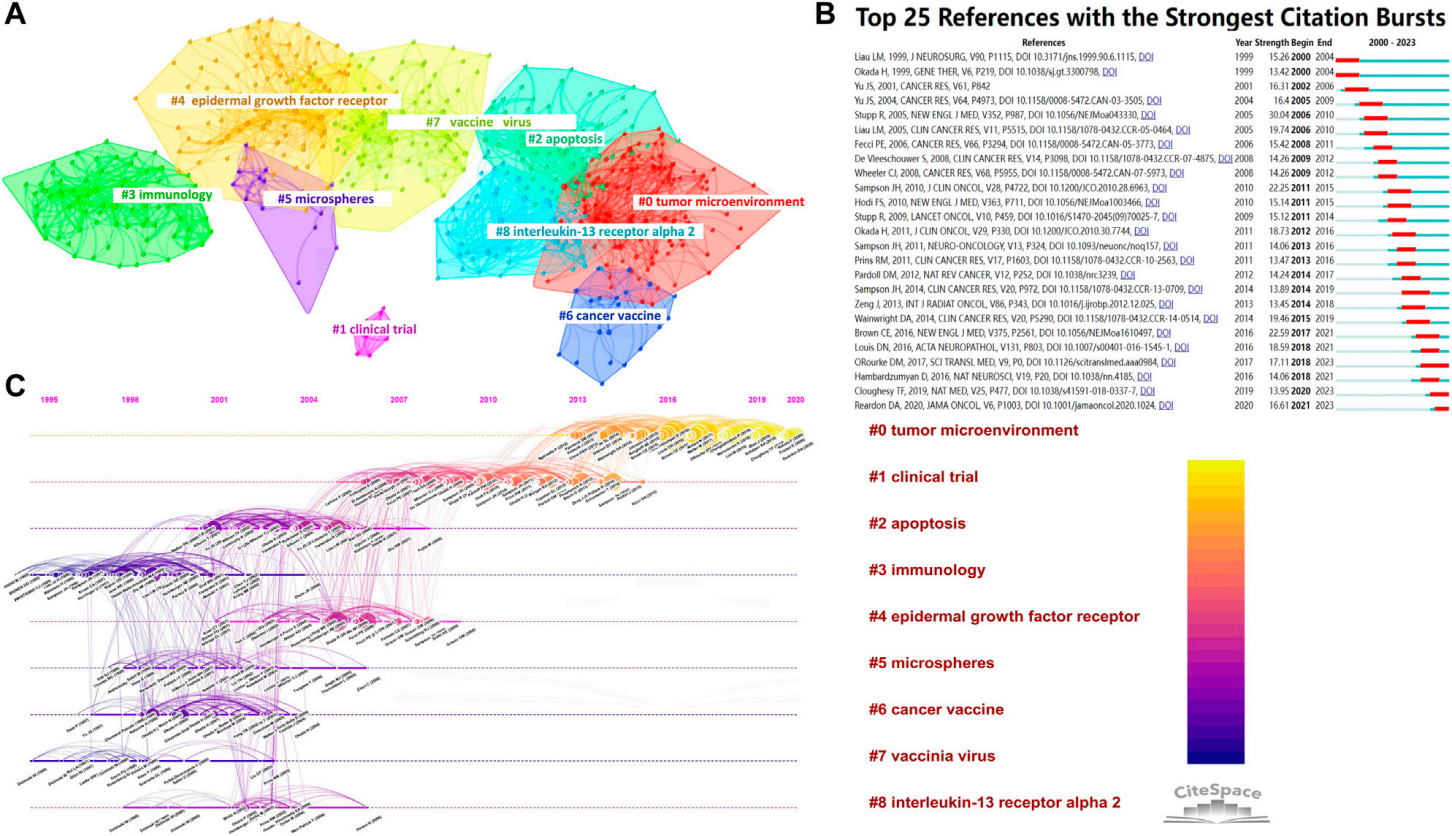
**(A)** Network visualization and clustering of co-cited references; **(B)** Top 25 references exhibiting citation bursts, arranged by the commencement year of the burst. The strength value indicates the intensity of the citation bursts, with red bars representing the duration; **(C)** Timeline visualization of co-citation analysis for references.

Between 1 January 2000, and 4 October 2023, out of the 54,395 references examined, 12,900 were co-cited. Supplementary Table S8 displays the top 20 co-cited references in terms of citations. Donald M. O'Rourke garnered the highest number of citations for his clinical trial article titled “A single dose of peripherally infused EGFRvIII-directed CAR T cells mediates antigen loss and induces adaptive resistance in patients with recurrent glioblastoma,” published in *Science Translational Medicine*. For enhanced visualization in CiteSpace, we configured the parameters to display co-citations, setting the time slice to 1 year, covering the period from 2000 to 2023, and focusing on the top 30% of cited references. Figure 8A depicts the co-cited reference network alongside a visualization cluster map. Each circle symbolizes a reference, with consistent colors indicating the same topic.

The results of our study show that the values achieved for modularity Q and mean silhouette S were 0.6678 and 0.886, respectively. The values indicate a significant clustering phenomenon and a remarkable level of uniformity. The data were organized into nine separate clusters, as shown in Figure 8A. Current research directions for cytokines on glioma immunotherapy mainly focus on #0 tumor microenvironment, #1 clinical trial, #2 apoptosis, #3 immunology, #4 epidermal growth factor receptor, #5 microspheres, #6 cancer vaccine, #7 vaccine virus and #8 interleukin-13 receptor alpha 2. The timeline display demonstrates the advancement of topic investigation, mapping the sequential path and length of co-citation growth in each cluster. For each year, the timelines showcase the most frequently referenced sources. Co-citation relationships are represented by lines that connect the nodes, and the clusters are organized vertically in a descending order based on their size. The colored curves represent co-citation connections that correspond to the year of each respective color. Nodes that are larger in size or have red tree-rings represent either bursts of citations, high counts of citations, or both. Figure 8C displays the arrangement of clusters along horizontal timelines in CiteSpace’s timeline visualization. This figure illustrates the distinctions within the nine clusters spanning from 2000 to 2023. The clusters are numbered from 0 to 8, with Cluster #0 being the biggest and Cluster #4 the second biggest. According to Figure 8B, certain clusters remained for a decade (until 2023), whereas others had a briefer lifespan. Figure 8B displays the prominent citation bursts from 2000 to 2023, illustrating the visual representation of the evolution and trend of cited references. Sorting is based on the year the burst commenced, with each year represented by a slice. The term “strength-value” indicates the intensity of the citation burst. Several reference bursts were identified in total. Research hotspots evolved over time. The earliest was “Liau LM, 1999,” emerging in 2000. “Reardon DA, 2020,” “Cloughesy TF, 2019,” “Hambardzumyan D, 2016,” “O'Rourke DM, 2017,” and “Louis DN, 2016” continued up to 2023, highlighting emerging areas of study, as depicted in Figure 8B.

## 4 Discussion

### 4.1 Publication trend

The rapid growth in publications on CGI in recent years indicates rising interest and activity in this research area. The exponential growth curve predicts continued growth, suggesting this will be an important area of focus going forward. The potential drivers of increased research interest in cytokines for glioma immunotherapy maybe consist in the following several aspects: Advances in cancer immunology and immunotherapy have spurred interest in harnessing the immune system against glioblastoma. Meanwhile, improved understanding of the glioma microenvironment is uncovering opportunities for cytokine-based strategies to overcome tumor immunosuppression. Technological developments like CAR T-cell therapies provide new tools for cytokine delivery (Lin et al., 2022). Increased recognition of the challenges in treating heterogeneous glioblastomas encourages the search for fresh immunotherapeutic approaches. Growing collaborations between neuro-oncologists and immunologists have brought new perspectives. Enhanced omics analyses of the tumor microenvironment enable more sophisticated evaluations of cytokine activity. Expanded funding and high unmet need for better glioblastoma therapies also contribute to rising interest in cytokine immunotherapy as an alternative, potentially improved treatment strategy. Further analysis of research and collaboration trends could provide additional insights into the factors stimulating increased activity focused on cytokines for glioma immunotherapies.

The predominance of articles over reviews suggests that this is still an emerging research area with many new findings being reported. As the field matures, we may expect to see more review publications synthesizing the state of the literature. The geographic distribution of publications reveals active research clusters in countries like the US, China, and Germany. Further bibliometric analysis of author affiliations and collaborations will be discussed later, which could shed light on connections between these research hubs. Tracking geographic trends over time can reveal the diffusion of CGI research around the globe. The recent rapid increase in publications provides opportunities for new synthesis and evaluation of the field. Bibliometric analysis provides helpful context for interpreting patterns in the CGI literature.

### 4.2 Countries

The United States and China hold dominant positions in cytokine research on glioma immunotherapy, with increasingly frequent collaborations between the two nations reflecting abundant research resources that could be further strengthened to promote disciplinary development. Although China ranks second in publication quantity, lower citation rates indicate Chinese scholars should enhance research quality to generate higher-impact outputs, through avenues like strengthened international partnerships, emphasized originality, and improved study designs. China should actively collaborate with leading Western countries to exchange talent and assimilate successful experiences for advancing its research capabilities. Emerging research powers like Iran and Slovenia are also progressively participating in this domain, providing opportunities for China to broaden perspectives through enhanced exchanges. Establishing international collaborative research centers could help congregate resources for rigorous multicenter clinical trials that elevate research quality. While international cooperation has grown substantially, ample potential remains based on bibliometric findings. Experts globally should increase communication to construct productive collaborative relationships and collectively propel advancements in this research field. Government agencies and funding bodies could institute special funds supporting international cooperative studies on glioma immunotherapy.

### 4.3 Institutions

The identified top 10 most productive institutions were primarily American, with just 3 Chinese centers represented, echoing the publication dominance of the United States in this research field. Bibliometric findings clearly demonstrate the substantial contributions and leading position maintained by American institutes like Duke University, the University of Pittsburgh, Harvard Medical School, and the University of California, Los Angeles. The high degree centrality exhibited by Duke University highlights its influence through well-connected and highly cited research constituting pivotal nodes that outline disciplinary progression. Capital Medical University ranked fifth for output volume as the top Chinese institute, underscoring China’s emerging significance. However, network analysis revealed the relatively low degree centralities and weaker connections of Chinese centers like Capital Medical University and Fudan University compared to American institutes. These findings indicate Chinese institutions should strengthen international cooperation through collaborative projects, academic exchanges, and engagement in global conferences to complement their existing research circles. Broader and deeper cooperative relationships can facilitate knowledge sharing between top Chinese and American institutes to the benefit of glioma immunotherapy research overall, while supporting the continued growth of China’s contributions. Focused efforts to build partnerships and participation in high-impact research could further enhance the position of Chinese institutions in this rapidly evolving field.

### 4.4 Authors

The identified prominence of American authors as the top contributors in glioma immunotherapy cytokine research highlights the leading role of the United States in this domain. Focused efforts to strengthen cooperative relationships between these influential American researchers and their subgroups could facilitate continued advancement and knowledge sharing. Bibliometric analysis spotlights authors like John H. Sampson and David A. Reardon as particularly impactful based on citation counts and h-indices, reflecting their pioneering contributions through high-volume, high-influence publications. Meanwhile, emerging authors from China and other nations with expertise in glioma immunotherapies should be integrated into these international collaborative networks to enrich perspectives. Co-authorship of publications involving experts across multiple countries could help disseminate knowledge and reinforce the global connections required to accelerate progress against this difficult disease. The symbiotic examination of co-citation and collaboration patterns provides a blueprint for reinforcing relationships between widely cited authors and identified research clusters. Strategic efforts to increase interconnectedness could pay dividends for the field.

### 4.5 Journals

Currently, source journals are concentrated in the fields of molecular biology and genetics, with less interdisciplinary linkage. However, based on projected citation pathways, emerging research areas are predicted in physics, materials science, chemistry, veterinary and animal science, clinical medicine, neurology, ophthalmology, psychology, education, and health sciences. Specifically, some new concepts and techniques in physical sciences and engineering, such as physics, materials science, and chemistry, may bring novel perspectives to this research field. For example, newly developed biomaterials and nanomaterials in materials science may provide better carrier options for immunotherapy. Additionally, advanced imaging technologies and high-throughput analytical techniques could also enable more precise experimental approaches in this field. From clinical medicine, neurology and other aspects, researchers may need to integrate interdisciplinary knowledge and view cytokine functions in the tumor microenvironment from a more comprehensive angle, establishing more accurate associations with disease progression and prognosis. This could facilitate translational medicine research in this field. Social sciences like psychology and education may also bring new research ideas to this field. For instance, how patients’ psychological status affects therapeutic efficacy, and how to improve patients’ cognition of and compliance with immunotherapy through health education.

In summary, these emerging potential interdisciplinary research directions provide great expansion opportunities for this field. Researchers should proactively engage in interdisciplinary collaboration and integration to propel further development of this research area. This study provides a comprehensive perspective on the publishing landscape, influential journals, projected research directions, and historical trends. The findings will inform researchers on impactful publication avenues and opportunities for cross-disciplinary collaboration in advancing cytokine research for glioma immunotherapies.

### 4.6 Keywords

79 keywords were analyzed in depth. The most frequent were “glioblastoma,” “immunotherapy,” and “T-cells”. The density of co-occurrences between keywords was visualized. The analysis shows how glioma immunotherapy research has progressed from an early focus on core therapies to a more nuanced understanding of the tumor environment and potential targets. The keywords and trends reflect the development of the field over time.

The keyword analysis provides valuable insights into the evolution of glioma immunotherapy research over time. The categorization of keywords into four main clusters reflects the multifaceted nature of this field. The first two clusters point to important developments in cytokine-based therapies and understanding cytokine regulation of the tumor microenvironment. The identification of core cytokine therapies like interleukin-13 and key mechanisms like TGF-beta modulation of immunosuppression reveals foundational areas of focus.

Meanwhile, the third and fourth clusters highlight the maturation of the field with keywords indicative of more sophisticated immunotherapeutic approaches. The activation of dendritic cells and focus on prime targets like angiogenesis denote an increased understanding of how to directly stimulate anti-tumor immunity. The keyword trends also mirror the progression of the field, with an increasing emphasis on illuminating the tumor microenvironment in recent years.

The burst analysis provides further evidence that glioma immunotherapy has built upon early groundwork in gene therapy and progressed to intricate concepts like the role of microglia. The frequency analysis showing “glioblastoma,” “immunotherapy” and “T-cells” as most common demonstrates the consolidation around key concepts.

Gene therapy: Another early hotspot, gene therapy represented an attempt to genetically modify tumor cells to make them more susceptible to immune-mediated killing. Strategies like introducing cytokines and costimulatory molecules into tumors helped establish important proof-of-concept evidence that modulating gene expression could render the tumor microenvironment more conducive to an antitumor immune response. “Malignant glioma”: Specifically focusing on malignant gliomas, the most aggressive primary brain tumors, was a sensible early emphasis. Glioblastoma is the most common and deadly glial malignancy. Defining its complex heterogeneity, invasive capacity, and resistance mechanisms was critical groundwork for the field. “Cytokines”: Cytokines represented logical early immunotherapeutic candidates. As key immune signaling proteins, directly augmenting cytokines like interferons and interleukins laid the groundwork for immunotherapy. Early insights into cytokine actions against gliomas proved an effective springboard. “Prognosis”: This hotspot points to research on predicting outcomes based on tumor and immune response markers. Prognosis reflects deeper understanding of how interactions between gliomas and immunity impact tumor progression and patient survival. “Inhibition”: Research on inhibitory mechanisms that suppress antitumor immunity expanded as the field grew more sophisticated. Checkpoint inhibitors targeting molecules like PD-1 emerged as key therapies, validating this as an important hotspot. “Resistance”: Gliomas exhibit primary and acquired resistance to immunotherapies, highlighting the need to unravel these mechanisms. Resistance remains a major challenge and hotspot for ongoing research. “Microglia”: Native CNS microglia play complex roles in gliomas, exhibiting both antitumor and immunosuppressive properties. Their contribution to the immunosuppressive microenvironment has made them an important recent hotspot. “Tumor microenvironment”: Beyond individual cells, the overall tumor microenvironment has risen as a central hotspot as researchers appreciate its multifaceted contribution to glioma progression and therapeutic resistance. Further unraveling its complexity is critical.

Overall, as shown through the keywords and their interconnectedness, glioma immunotherapy has clearly advanced considerably from preliminary attempts to modulate the immune system to an intricate and multi-faceted approach targeting specific mechanisms and cell types. The study provides a quantitative understanding of this evolution. Future research may benefit from focusing on undersaturated nodes in the keyword network as well as continuing to explore emerging topics like the tumor microenvironment.

### 4.7 Literatures Co-citation

The co-citation analysis provides a quantitative mapping of the knowledge structure and research evolution of cytokines for glioma immunotherapy over the past 2 decades. Clustering the most co-cited references reveals the main research directions in this field, with tumor microenvironment and EGFR as the largest clusters, indicating their importance as core research foci. The timeline visualization clearly traces the rise and fall of research topics, with some persisting over long periods while others were short-lived bursts of activity. Tracking the citation bursts quantifies the historically most impactful contributions, from foundational early works like Liau 1999 to recent advances like Reardon 2020 that point to emerging subfields.

This scientometric mapping at a macro-level reveals the research landscape and developmental trajectory of this field. The findings could inform researchers on influential historical works to build upon, understudied areas warranting more investigation, and projected impactful directions to pursue next. Researchers should consider opportunities to integrate knowledge across the clusters through cross-disciplinary collaboration. For example, synergizing insights from tumor microenvironment, immunology, and clinical trials research may accelerate translation of basic cytokine biology findings into clinical applications. Strategic collaboration across specialties and institutions can help unify and strengthen the field. The quantitative mapping of scientific progress over time provides an evidence base to guide future discovery and innovation in cytokines for glioma immunotherapy.

#0 Tumor Microenvironment (Barthel et al., 2022): The tumor microenvironment cluster highlights the importance of studying cytokine signaling and immune dynamics in the context of the complex glioma microenvironment. Key knowledge gaps exist regarding how various immune components interact and are modulated by cytokines and other signals from cancer and stromal cells. Further research should aim to unravel these complex dynamics through high-dimensional profiling and integrative modeling; #1 Clinical Trials (Chen et al., 2017): The clinical trials cluster underscores the need to translate cytokine biology findings into human studies. As a core pillar of glioma immunotherapy research, well-designed trials are essential to systematically evaluate safety, efficacy, and biomarkers; #2 Apoptosis (Chang et al., 2021): The apoptosis cluster suggests that delineating cytokine regulation of cell death pathways in glioma remains pivotal. Therapeutically inducing glioma apoptosis through cytokine signaling may overcome resistance. Further mechanistic work should clarify how cytokine-mediated apoptosis interconnects with autophagy, necrosis, and other death modalities in glioma; #3 Immunology: The immunology cluster highlights active investigation into modulating anti-glioma functionality of immune cell types via cytokines; #4 Epidermal Growth Factor Receptor: The EGFR cluster indicates this remains a key target for cytokines and immunotherapy given its frequent dysregulation in glioma. Studies should continue elucidating mechanisms of cytokine-mediated EGFR inhibition and related therapeutic resistance. Combining EGFR- and cytokine-targeted therapies may improve outcomes; #5 Microspheres: The microspheres cluster suggests drug delivery systems are important to optimize cytokine delivery and release kinetics; #6 Cancer Vaccines: The cancer vaccines cluster highlights their rising potential to stimulate anti-glioma immunity through cytokine activation. Combining cytokine adjuvants with tumor antigens may improve vaccine potency; #7 Vaccine Viruses: This cluster indicates oncolytic viruses are gaining promise as novel cytokine delivery platforms. Combining cytokine transgene expression with viral oncolysis may amplify antitumor immune responses; #8 Interleukin-13 Receptor Alpha 2: The IL13Rα2 cluster suggests this glioma antigen is a promising target for cytokine-mediated immunotherapy. Studying IL13Rα2-targeted cytokine delivery and related immune effects can refine targeting specificity. The clustered research directions provide a roadmap for progress across key facets of cytokine immunotherapy for glioma. Targeting these areas through cross-disciplinary collaboration promises to advance the field towards improved patient outcomes.

### 4.8 Overview of cytokines in glioma immunotherapy

Cytokines are low molecular weight proteins or peptides synthesized and secreted by a variety of cells in response to immunogen, mitogen or other stimuli (Liu et al., 2021). They have a wide range of biological activities and can mediate signal transduction and interaction between cells. Cytokines play an important role in innate and adaptive immune responses and can nonspecifically stimulate the activation, proliferation and differentiation of T cells (Zeng et al., 2021).

#### 4.8.1 Classification of cytokines

According to their main functions, cytokines can be divided into six categories.

##### 4.8.1.1 Interleukin

Interleukin (IL) family is a kind of cytokine that regulates the immune system bidirecally and participates in the differentiation and activation of immune cells. IL can be divided into IL-1 family, IL-10 family, IL-12 family, etc (Tavakolpour et al., 2020).

##### 4.8.1.2 Interferon

Interferon (IFN) is a protein or glycoprotein produced by the body when stimulated (Milanés-Virelles et al., 2008). It can be divided into three categories: IFN-α, IFN-β and Ifn-γ. Type I IFN is mainly produced by innate immune cells (Kim, 2021). Type II IFN is mainly produced by innate and acquired immune cells (Banchereau et al., 2017). IFN can synergistically promote immune responses and have antiviral, antitumor and immunomodulatory functions (Li et al., 2007).

##### 4.8.1.3 Tumor necrosis factor

Tumor necrosis factor (TNF) is a kind of pro-inflammatory cytokines, including Tnf-α and TNF-β, which are mostly produced by immune cells (Weersma et al., 2007). High expression of TNF-α can activate T cells and promote the production and secretion of a variety of cytokines, thereby causing inflammatory responses such as fever and macrophage aggregation (Cruceriu et al., 2020).

##### 4.8.1.4 Colony-stimulating factor

Colony-stimulating factor (CSF) can promote the proliferation and differentiation of hematopoietic progenitor cells (Stachura et al., 2013). It is a component of the proinflammatory cytokine network and participates in the inflammatory process. CSF can upregulate the number of macrophages in the inflammatory site, leading to the persistence and amplification of inflammation (Liu X. et al., 2022).

##### 4.8.1.5 Chemokines

Chemokines are a class of low molecular weight proteins that are divided into four subfamilies based on their amino acid sequence (Uza et al., 2011). Different subfamilies of chemokines can specifically regulate the movement and homing of various immune cells and participate in a variety of physiological processes. Some chemokines are pro-inflammatory while others serve to maintain homeostasis (Bikfalvi and Billottet, 2020).

##### 4.8.1.6 Growth factors

Growth factors, such as IGF, EGF, and TGF-β, are peptides that can affect cell growth, differentiation, apoptosis and immunity (Almodóvar et al., 2014). Different growth factors regulate cell functions through various pathways and are widely involved in physiological processes (Haque and Morris, 2017).

#### 4.8.2 Roles of several important cytokines in glioma immunotherapy

##### 4.8.2.1 IL

**FIGURE 9:**
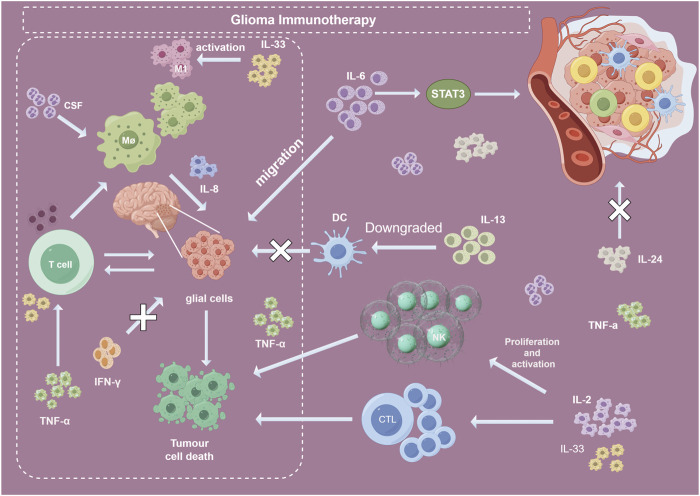
Roles of several important CGI. Abbreviation: CGI, cytokines in glioma immunotherapy; IFN, interferons; IL, interleukin; TNF, tumor necrosis factors; MΦ, macrophage; DC, dendritic cell; NK, natural killer; CSF, Colony-stimulating factor; CTL, cytotoxic T lymphocytes.

Interleukin-2 (IL-2) has been extensively studied for the immunotherapy of gliomas (Liu et al., 2017). IL-2 can significantly promote the proliferation and activation of T cells, particularly CD8^+^ cytotoxic T cells (Smith et al., 2017), which can directly kill tumor cells. Furthermore, as shown in the Figure 9, IL-2 may help maintain the population of CD4^+^ helper T cells (Liu Y. et al., 2021), which are critical for coordinating the overall antitumor immune response. Additionally, IL-2 can mediate the proliferation and activation of natural killer (NK) cells (Kong et al., 2017), enhancing their tumor lytic capabilities. IL-2 has also been demonstrated to drive the sustained expansion and survival of cytotoxic T lymphocytes (CTLs) (Rollings et al., 2018). In summary, while appropriate doses of IL-2 remain an important component of glioma immunotherapy, IL-2 needs to be used in combination with other immunotherapeutic strategies to maximize synergistic therapeutic effects.

IL-6 is highly expressed in human glioma specimens and cell lines, and activates the transcription of multiple tumor suppressor genes through STAT3 (signal transducer and activator of transcription 3) signaling pathway to promote the proliferation and inhibit apoptosis of U251 and A172 cell lines. Studies have shown that IL-6 can promote the migration of glioma cells (Wang et al., 2019), and cucuritin-1, a specific inhibitor of STAT3, can inhibit this promoting effect of IL-6 (Lin et al., 2011), suggesting that IL-6 may regulate the invasion of glioma through JAK/STAT pathway. In addition, IL-6 promoted tumor angiogenesis and tumor growth by activating STAT3 in glioma. A previous study reported that elevated circulating levels of IL-6, IL-8, IL-17, TNF-α, TGF-β, and CRP are significantly linked to an increased risk of glioma. Furthermore, circulating IL-6 and CRP have the potential to act as potent prognostic biomarkers for unfavorable outcomes in glioma patients (Feng et al., 2019). Further study of the downstream signaling pathways of IL-6 will help to understand its role in glioma and its clinical application.

Interleukin-8 (IL-8) is an important proinflammatory chemokine and key regulator in the tumor microenvironment (Orlikova and Diederich, 2012). Specifically, IL-8 can promote the recruitment and activation of immunosuppressive granulocytes in glioma tissues (Törnblom et al., 2019), which inhibits the cytotoxic activity of effector T cells against tumor cells. A study by Iglesia et al. using U87 and A172 human glioma cell lines with tumor suppressor gene deletions found that phosphorylated STAT3 molecules transcriptionally repressed IL-8 by occupying its promoter in the nucleus, thereby inhibiting tumor cell proliferation and invasion (de la Iglesia et al., 2008). Knocking out IL-8 expression via RNA interference in U87 cells inhibited proliferation and invasion, indicating IL-8 can promote glioma cell proliferation and invasion.

##### 4.8.2.2 IFN

Interferon (IFN) plays a pivotal role in the immunotherapy of glioma (Li et al., 2020). IFN can directly inhibit glioma cell proliferation (Pasquali and Mocellin, 2010) and enhance anti-tumor immune responses through multiple pathways (Li et al., 2017). It upregulates major histocompatibility complex (MHC) molecule expression on glioma cells, enhancing their immunogenicity (Giannandrea et al., 2009; Ahmed et al., 2018). *In vitro* studies show IFN-γ strongly inhibits human glioma cell proliferation and reduces hyaluronic acid adhesion (Knüpfer et al., 1997). Besides IFN-γ, IFN-α also has immunotherapeutic potential in glioma. Intramuscular injection of plasmid DNA encoding mouse IFN-α exerts potent anti-tumor effects against primary and metastatic tumors like glioma and melanoma in mice (Horton et al., 1999). In summary, IFN plays dual anti-tumor and immunotherapeutic roles in glioma by coordinately regulating immune responses, and is a key immunomodulator for glioma immunotherapy.

##### 4.8.2.3 TNF

TNF directly induces apoptosis and necrosis in tumor cells (Adjuto-Saccone et al., 2021). It elevates the expression of death receptors on tumor cell surfaces, binding to them to activate downstream apoptotic pathways (Zhang et al., 2020). Furthermore, TNF enhances the cytotoxic activity of immune effector cells, such as macrophages and NK cells, against tumor cells (Müller and Kontermann, 2010). It also promotes apoptosis in tumor vascular endothelial cells and suppresses tumor neovascularization (Van Hauwermeiren et al., 2013). In glioma immunotherapy, TNF is predominantly utilized in gene therapy (Wang et al., 2012). Fukushima et al. conducted a study with 17 malignant astrocytoma patients. They divided the participants into two groups: the MR Group received ranimustine chemotherapy (MCNU) combined with radiotherapy, while the TMR Group received the same treatment plus recombinant human tumor necrosis factor-alpha (TNF-SAM2). The findings indicated that anaplastic astrocytoma patients in the TMR Group had a longer survival rate than those in the MR Group. This suggests a potential benefit in combining chemotherapy and radiotherapy with TNF-SAM2 for anaplastic astrocytoma patients, although the sample size was limited (Fukushima et al., 1998). In conclusion, TNF modulates the anti-tumor immune response through various mechanisms, establishing its significance in glioma immunotherapy.

##### 4.8.2.4 CSF

Colony-stimulating factor (CSF) is a crucial hematopoietic growth factor that fosters the proliferation and differentiation of various white blood cell lineages, underscoring its significance in glioma immunotherapy. CSF facilitates the differentiation of bone marrow stem cells into immune effector cells, including dendritic cells, macrophages, and natural killer cells, thereby bolstering the body’s overall immune function (Shi et al., 2006). Moreover, CSF directly enhances the activity and anti-tumor capabilities of these immune cells. For instance, CSF fosters the maturation of dendritic cells, augmenting their antigen-presenting capacity and boosting cytotoxicity by activating natural killer cells and macrophages (Palata et al., 2019). Additionally, CSF synergizes with other cytokines, such as IL-24, to amplify the body’s tumor immune response (Deng et al., 2020). In summary, CSF elevates the body’s immune status and anti-tumor efficacy through various mechanisms, solidifying its role as a pivotal immunomodulatory factor in glioma immunotherapy.

##### 4.8.2.5 Chemokines

Chemokines are a class of small molecule cytokines that can specifically recruit immune cells and play an important role in the immunotherapy of gliomas (Nong et al., 2023). Chemokines can promote the directional migration and aggregation of various immune effector cells to tumor tissues (Do et al., 2020). For example, CXCL10 can promote the recruitment of T lymphocytes and natural killer cells (Pociask et al., 2011); CCL2 can promote the aggregation of monocytes and macrophages (Loomis-King et al., 2013). In addition, some chemokines can directly enhance the activity of immune cells (Glodde and Hölzel, 2017). In the immunotherapy of glioma, the application of exogenous chemokines can significantly increase the infiltration of immune cells at the tumor site and enhance the antitumor immune response (Song et al., 2022). In conclusion, chemokines are important links that connect and amplify the systemic and local immune systems of the body, which is an effective method to optimize the immunotherapy of glioma.

##### 4.8.2.6 Growth factors

Growth factors serve bifunctional roles in tumor immunotherapy (Miller et al., 2008; Naber et al., 2012). While certain growth factors, such as EGF and FGF, can stimulate glioma cell proliferation (Karl et al., 2008), high VEGF expression is crucial for glioma angiogenesis (Yamanaka and Saya, 2009). Overexpressing these factors can compromise the anti-tumor immune response. Conversely, some growth factors, like GM-CSF, amplify the antigen-presenting activity of dendritic cells and macrophages and heighten macrophage cytotoxicity against tumor cells (Triozzi et al., 2005). Therefore, in glioma immunotherapy, interventions should be tailored based on the distinct functionalities of these factors. Strategies should encompass inhibiting the expression and activity of tumor-promoting growth factors while judiciously employing those with immune-enhancing properties. Merely inhibiting tumor growth is insufficient; optimizing the body’s immune status is vital to enhance the efficacy of glioma immunotherapy.

The potent immunosuppressive cytokine TGF-beta has also emerged as an attractive target for glioma immunotherapy. TGF-beta facilitates immune evasion and cancer progression through promoting epithelial-mesenchymal transition (EMT) and inhibiting functions of lymphocytes. Targeting TGF-beta therefore offers dual benefits by reversing immunosuppression and reducing invasiveness. Recently, bispecific antibodies simultaneously targeting TGF-beta and the immune checkpoint PD-L1 have shown promising antitumor effects. The lead asset YM101 exhibits subnanomolar affinity for human TGF-beta and PD-L1, potent TGF-beta neutralization, and T cell activation. Preclinical studies of YM101 demonstrated robust T cell proliferation, cytokine production, and cytolytic activity against glioma cells. Another bispecific antibody termed BiTP, targeting murine TGF-beta and PD-L1, showed survival benefits in glioma mouse models through increased tumor immunogenicity and cytotoxic T cell infiltration.

These pioneering anti-TGF-beta/PD-L1 bispecific antibodies exemplify a synergistic approach harnessing blockade of immunosuppression and reinvigoration of anti-tumor immunity. Further optimization of affinity, half-life, and effector functions should enable clinical translation. As the glioma immune microenvironment exhibits abundant extracellular TGF-beta, ongoing Phase I trials of YM101 and related bispecific antibodies may unlock exceptional responses beyond anti-PD-L1 monotherapy. Altogether, joint cytokine and checkpoint immunotherapies represent a promising frontier in the continuing search for glioma treatment advances.

### 4.9 Strengths and limitations

This bibliometric analysis provides the first quantitative mapping of the research landscape on cytokines for glioma immunotherapy. Notably, bibliometric analysis offers a more comprehensive perspective than traditional literature reviews and provides enhanced visualization. The findings offer scholars an evidence-based guide to recognize influential contributors, publications, and research directions in this domain. Illuminating the rising focus on intricacies of the glioma microenvironment will help scholars shape investigations. Identifying understudied areas provides opportunities for scholars. Historic research patterns clarify current developmental contexts to inform scholarly inquiry. The analysis spotlights cross-disciplinary collaboration as key for scholars to collectively advance glioma immunotherapies. This macro-level visualization of the glioma immunotherapy cytokine research topography acts as an invaluable strategicorienting resource for scholars worldwide. In addition, various software tools were employed in this bibliometric analysis for data analysis and visualization, including Microsoft Excel 2021, Origin 2023, Microsoft Charticulator, VOSviewer 1.6.19, Citespace 5.7R3, and the Bibliometrix package in RStudio. By utilizing these tools, a thorough comprehension of the pertinent literature, focal points of research, and current patterns concerning the impact of cytokines on glioma immunotherapy was facilitated. There are certain constraints to this research. Initially, solely papers written in English from the WoSCC database were incorporated; forthcoming research should examine additional databases. Moreover, the bibliometric evaluation was limited to metadata instead of the complete text, which may result in the omission of important findings exclusively present in the articles. Given the ongoing updates to the database, this analysis covers pertinent records starting from 1 January 2000, until 4 October 2023.As a result, there could be variations between these discoveries and the latest research on CGI.

## 5 Conclusion

In our groundbreaking bibliometric analysis of CGI literature spanning 1 January 2000, to 4 October 2023, we noted a marked increase in research activity, with the United States taking the lead. Future research will likely focus on the tumor microenvironment, cancer vaccines, epidermal growth factor receptor, and interleukin-13 receptor alpha 2. Glioma immunotherapy will continue to develop through investigations into resistance mechanisms, microglia and macrophage biology, and interactions within the complex tumor microenvironment. Enhanced global collaboration is vital for advancements in these domains.
