# Supplementary material for: Unveiling the landscape of cytokine research in glioma immunotherapy: a scientometrics analysis
Source: Front Pharmacol. 2024 Jan 8;14:1333124. doi: 10.3389/fphar.2023.1333124 (PMC10800575; doi:10.3389/fphar.2023.1333124)
Supplement: Supplementary file 1 [file DataSheet2.pdf]

Supplementary Material

Supplementary Tables

Supplementary Table 1. Number of publications per year from the top 10 countries.

Supplementary Table 2. The number of publications of SCP and MCP in the top 10 countries by volume.

Abbreviation: SCP refers to Single Country Publications, where all authors hail from the same nation. Conversely, MCP stands for Multiple Country Publications, denoting articles with authors from different countries, signifying international collaboration.

Supplementary Table 3. Radar chart of the top five institutions in terms of publications.

Abbreviation: ACPP, Average number of citations per paper.

Supplementary Table 4. The number of papers published per year by the top ten authors.

Supplementary Table 5. Top 10 co-cited journals in citations.

Supplementary Table 6. The annual number of publications of the top five journals.

Supplementary Table 7. Cytokine research for glioma immunotherapy in journals with high impact factors (IF 2022>20).

Abbreviation: IF, impact factor.

Supplementary Table 8. Top 20 co-cited references in citations

Supplementary Table 9. 1,529 publications from the WOS.

Abbreviation: WOS, Web of Science.
